# A computational model for understanding the micro-mechanics of collagen fiber network in the tunica adventitia

**DOI:** 10.1007/s10237-019-01161-1

**Published:** 2019-05-07

**Authors:** Venkat Ayyalasomayajula, Baptiste Pierrat, Pierre Badel

**Affiliations:** Mines Saint-Étienne, Univ Lyon, Univ Jean Monnet, INSERM, U1059 SAINBIOSE, Centre CIS, 42023 Saint-Étienne, France

**Keywords:** Tunica adventitia, Finite elements, Rupture, Collagen fibers

## Abstract

Abdominal aortic aneurysm is a prevalent cardiovascular disease with high mortality rates. The mechanical response of the arterial wall relies on the organizational and structural behavior of its microstructural components, and thus, a detailed understanding of the microscopic mechanical response of the arterial wall layers at loads ranging up to rupture is necessary to improve diagnostic techniques and possibly treatments. Following the common notion that adventitia is the ultimate barrier at loads close to rupture, in the present study, a finite element model of adventitial collagen network was developed to study the mechanical state at the fiber level under uniaxial loading. Image stacks of the rabbit carotid adventitial tissue at rest and under uniaxial tension obtained using multi-photon microscopy were used in this study, as well as the force–displacement curves obtained from previously published experiments. Morphological parameters like fiber orientation distribution, waviness, and volume fraction were extracted for one sample from the confocal image stacks. An inverse random sampling approach combined with a random walk algorithm was employed to reconstruct the collagen network for numerical simulation. The model was then verified using experimental stress–stretch curves. The model shows the remarkable capacity of collagen fibers to uncrimp and reorient in the loading direction. These results further show that at high stretches, collagen network behaves in a highly non-affine manner, which was quantified for each sample. A comprehensive parameter study to understand the relationship between structural parameters and their influence on mechanical behavior is presented. Through this study, the model was used to conclude important structure–function relationships that control the mechanical response. Our results also show that at loads close to rupture, the probability of failure occurring at the fiber level is up to 2%. Uncertainties in usually employed rupture risk indicators and the stochastic nature of the event of rupture combined with limited knowledge on the microscopic determinants motivate the development of such an analysis. Moreover, this study will advance the study of coupling microscopic mechanisms to rupture of the artery as a whole.

## Introduction

Aortic aneurysm can be identified as a focal dilation of the blood vessel in comparison with the normal, healthy artery (Aggarwal et al. 2011; Humphrey and Holzapfel 2012). Aortic aneurysms are found to fail in two ways: dissection and rupture, mechanical phenomena that occur when the wall stress exceeds the local strength of the artery (Humphrey and Taylor 2008). This fatal condition has been reported to have a mortality rate of up to 85% in western countries (Erhart et al. 2014; Robert et al. 2017). Maximum vessel diameter and diameter increase rate are the clinical indicators used for decision making. Peak wall stress is also an indicator often promoted for predicting rupture (Marini et al. 2012; Gasser et al. 2010). However, various cases have been recorded where the above indicators fail to predict aortic rupture. This might be due to the fact that these indicators are based on the macroscopic mechanical state of the tissue and do not take into consideration the mechanics at the micro-level, and the local strength of the tissue. Hence, in order to better understand the mechanical phenomena responsible for rupture, an accurate description of the mechanical response at the level of the microstructure is needed. Structurally accurate computational models of arterial wall layers are still an improving aspect of arterial mechanics and any step forward would be very significant.

**Fig. 1:**
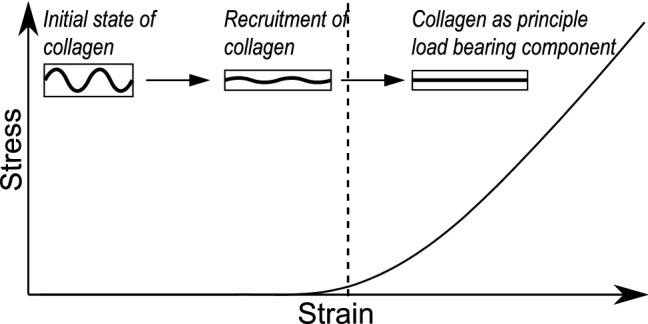
Mechanical behavior of collagen under uniaxial tension

The current paper focuses on the tunica adventitia, which is the outermost layer of the arterial wall. The adventitia primarily consists of a dense network of collagen type I fibers, a small amount of elastin fibers, fibroblasts, nerves, vasa vasorum, and connective tissue (He and Roach 1994). The collagen fibers in the adventitia tend to have a complex organizational structure with highly dispersed orientations and undulations in their unloaded configuration (Smith et al. 1981). In contrast, the fibers in the tunica media, the middle layer, are less undulated and highly aligned toward the circumferential direction (Wolinsky and Glagov 1967). The distribution and organization of collagen in the adventitia gives the layer its unique structural integrity. The adventitia serves various functions like anchoring the wall to the surrounding tissue, supplying necessary nutrients and oxygen through vasa vasorum, and synthesizing collagen through fibroblasts. But, the main function of the adventitia is to protect the arterial wall from overdistention and rupture, i.e., is to act as a protective sheath at loads beyond physiological pressure (Humphrey 2002). This is facilitated by the fact that the elastic modulus of collagen fibers is much higher than that of elastin (Oxlund and Andreassen 1980; Viidik et al. 1982). As the pressure increases, collagen fibers in the adventitia get straightened and become stiff to carry the load. Hence, the overall tissue mechanical response is highly dependent on the microscopic structural organization of collagen as illustrated in Fig. 1.

Numerical models provide an alternate means to study the evolution of abdominal aortic aneurysm and understand rupture as they are noninvasive and time-efficient. Initial advances in developing a computational model for the arterial wall gave rise to the realization that, due to the nonlinear, anisotropic stress–strain behavior, mathematical descriptions in the form of exponential, polynomial, or logarithmic functions are needed to extrapolate the experimental results to full range of in vivo loading conditions (Wagenseil and Mecham 2009). This type of modeling technique where a strain energy density function is deployed to compute stresses is called phenomenological modeling. Phenomenological models vary in terms of their choice of strain energy density function and the number of parameters. These parameters are later fitted using nonlinear regression methods with a comparison to experimental data. Some of the widely used phenomenological models include: seven-parameter polynomial (Vaishnav et al. 1973), four-parameter logarithmic model (Takamizawa and Hayashi 1988), four- or seven-parameter exponential models (Chuong and Fung 1983). Humphrey made a detailed comparative study of the strain energy functions proposed in above-mentioned literature (Humphrey 1999). Although phenomenological models predict the behavior of the tissue accurately, they do not account for any structural information. This leads to difficulties in studying and predicting the behavior of diseased arteries where changes in physiology and pathology directly influence the mechanical behavior (Alford et al. 2008).

On the other hand, structurally motivated constitutive models incorporate the contributions of individual microstructural components in the strain energy density function (Zulliger and Stergiopulos 2007). A number of researchers investigated structural constitutive models, which can be broadly classified into two categories: the angular integration approach and the structural tensor approach (Gasser et al. 2006). Improving on their existing physiological exponential model (Holzapfel and Weizsäcker 1998) used for predicting the behavior of arterial walls in the physiological pressure range, Holzapfel et al. proposed a structural model for individual layers of the arterial wall. Two structural tensors each representing a family of collagen fibers are used to model the anisotropic behavior of collagen. Collagen is assumed to be embedded as parallel fibers within each family, which is limited by the fact that collagen is dispersed in the arterial wall and does not maintain a fixed orientation at no-distention state. A generalized structure tensor was introduced by Gasser et al. (2012) to address the above limitation. More recently, based on their experimental findings, a bivariate von Mises distribution to define the structural tensor was implemented in Polzer et al. (2013). Angular integration approaches, on the other hand, utilize a probability distribution for the fibers to be incorporated directly into the strain energy potential (Gasser et al. 2006; Holzapfel et al. 2014). In Alastrué et al. (2010), a Bingham distribution for collagen was used to statistically model the mechanical response of the arterial wall within the angular integration framework. A detailed study made in Holzapfel et al. (2015) and Cortes et al. (2010) to evaluate these two approaches concluded that both approaches yield significantly different results at high fiber dispersion and for different loading conditions. Although these formulations are straightforward and offer significant computational advantages, the parameters are often derived using an inverse approach rather than a histology study, like in purely phenomenological approaches. In addition, the structural organization arising from crimp is not directly considered from such analysis.

Another important structural aspect of fibrous tissues is the presence of waviness and the phenomenon of fiber recruitment. A few of the existing microstructural constitutive models assume a wave shape for the collagen crimp and incorporate it at either the fibril or molecular level. Lanir (1979, 1983) pioneered this work by laying out the theoretical framework for modeling collagen fiber as a two-dimensional wave, with statistical distributions for either the wave shape or crimp behavior. This work was followed by the idea of modeling collagen crimp as a stochastic parameter (Cacho et al. 2007) and motivated further works which adopted it for constitutive modeling of various fibrous soft tissues (Zulliger and Stergiopulos 2007; Hurschler et al. 1997). Assuming a wave-like structure for the shape of crimped collagen fibers was also considered for discrete models by introducing kinematic linkages between small straight fiber segments (Stouffer et al. 1985; Kastelic et al. 1980; Diamant et al. 1972). Owing to the ambiguity in observing the shape of collagen crimp from experiments, Beskos and Jenkins (1975) proposed a cylindrical helix to represent it and derived a constitutive model based on this assumption. Being the first to model the collagen crimp as a smooth 3-D wave, the model was riddled with limitations, one of which was the assumption of fiber in-extensibility resulting in an infinite stiffness at full extension. Several authors extended the model by modifying the fiber response with simple (Freed and Doehring 2005) or more refined assumption like that of beam deformation (Grytz and Meschke 2009). These models were shown to be capable of reproducing stress–stretch responses of fibrous tissues in uniaxial tension, but their outputs in terms of microstructural geometry were not completely in agreement with experimental observations. In this context, the advantage of a discrete numerical model would be to consider the kinematics of fibers at a local and individual level, thus enabling non-affine transformation, and possible local interactions. Multi-scale homogenization method constitutes another class of models to be mentioned. They incorporate collagen crimp at either the fibrillar level or the fiber level also as a function of strain (Marino and Vairo 2014; Bianchi et al. 2016). In the framework of arterial mechanics, these models have provided novel insights in various scenarios, especially in rupture prediction, however, they often assume an average value of the constitutive parameter for the whole wall, thus neglecting local heterogeneities. Discrete models are suitable to address this limitation and motivated the presented work.

Another way of studying micro-mechanics of homogenous fibrous networks is to incorporate the network geometry into the model. One approach for incorporating structural information of the fiber network can be achieved through image processing, in which the desired network is segmented from the images and skeletonized (Krauss et al. 2012). Instead, numerically generated random fiber networks have been instrumental in studying the mechanics of large network models like collagen gels, carbon nanotubes, polymer networks, etc. (D’Amore et al. 2010; Spanos and Esteva 2009; Lake et al. 2012; Zhang et al. 2012). The mechanical behavior of human amnion was modeled and predicted using a discrete network model by ensuring geometric and stiffness percolation (Mauri et al. 2016; Bircher et al. 2017). Commonly used methods like Voronoi tessellations, Delaunay triangulations and other tessellations resulting from similar construction principles are not ideal for studying complex structures such as the adventitia. This is due to the fact that even though the structural arrangement of fibers in the adventitia is random, it can be identified by a stochastic process with proper geometrical implications. This is necessary as the mechanical response of the structure is highly sensitive to the microscopic arrangements of the fibers. More recently, random walk algorithms have been used to model fiber networks in fibrous biomaterials and arterial wall layers (Jin and Stanciulescu 2016). Although the approach presented by Jin and Stanciulescu (2016) utilized morphological parameters like orientation and waviness to describe the fiber network, the model fails to address some important aspects related to morphology and kinematics. Firstly, the assumption of realizing the collagen network to be made up of two families of fibers with identical mean orientations about the axis with an equal percentage of fibers in both families for adventitia. Upon investigation of several second-harmonic generation microscopy image stacks of adventitia, we observed that this is not the case where sometimes even the presence of two families of fibers is indistinguishable. Secondly, the assumption that the underlying kinematics of the fibers follow an affine transformation is perhaps inaccurate. Random fiber networks such as collagen in the adventitia are shown to not necessarily undergo affine kinematics (Krasny et al. 2017; Chandran 2005). These factors pose some important questions to be addressed, which motivated us in developing a discrete model generated based on experimentally measured orientation distributions, and including features to enable and control non-affinity of the networks transformation, similar to the previous works (Mauri et al. 2016).

Hence, the main objective of this paper is to bridge the gap by combining the above-mentioned numerical reconstruction methods with a thorough histological analysis and stochastic quantification of the microstructure, with the aim to study the microscale mechanical state of such fibrous tissues. The interactions between neighboring fibers, and between fibers and the ground matrix are not included in the model. The possible contribution of the matrix on the mechanical response of the tissue is in the form of hydrostatic pressure developing with stretch (Lanir 1979). The assumption to exclude any interactions between the ground matrix and fibers was shown to be a valid one by some studies (Harkness and Harkness 1959; Daly 1969). The present study was based on experimental data previously obtained on rabbit carotid arteries using multi-photon confocal microscopy and uniaxial tension testing (Krasny et al. 2017). Herein, we hypothesized that the parameters which influence the passive mechanics of the adventitial fiber network are: orientation, waviness, volume fraction, diameter and elastic modulus of the fibers. Note that the study was limited to the mechanics of the collagen network in the adventitia most relevant in studying extreme loading scenarios neglecting the effects of other microstructural components. This choice lied onto the common hypothesis that at high loads beyond physiological pressure levels, collagen acts as the principal load-bearing component in the arterial wall. Through thorough histological analysis, the morphological parameters were quantified for one sample. Next, discrete network modeling was implemented for reconstructing the collagen fiber network which was further used as an input for numerical (Finite Element, FE) simulations of uniaxial tension tests. A constrained optimization problem was defined to identify constitutive (and morphological, if missing) parameters for various samples. After finding a good agreement for the macroscopic mechanical behavior, the microscopic mechanical state was quantified and analyzed. Finally, an extensive sensitivity analysis was performed to establish the relationship between (1) the selected mechanical outputs and (2) each of the governing constitutive/morphological parameters, thus providing insight to discuss structure-to-mechanics relationships in adventitial tissue. A brief discussion on the non-affine nature of fiber kinematics is provided based on the obtained results. And finally, utilizing the result of the mechanical state of the fibers at various load levels, a possible probabilistic tool for understanding the onset of rupture is presented.

## Methods

### Experimental data and analysis

All the data that were used to develop and validate the finite element model were acquired from experiments on carotid arteries from male New Zealand White rabbits which were previously published (Krasny et al. 2017). Briefly, the initial pre-stretch condition of the excised samples was assessed by measuring in vivo and ex vivo lengths promptly after harvesting. The excised samples were frozen until the day of the experiment at $-\,20\,^{\circ }$C and then unfrozen in a phosphate-buffered saline solution at an ambient temperature of $24\,^{\circ }$C. It should be noted that the mechanical tests were conducted at a temperature less than the one in vivo, which might possibly stiffen the arterial wall (Zemanek and Michal 2009). Also, it was evident from the literature that freezing the arteries also induces mechanical and structural changes, but there is no consensus as to what kind of histological changes happen (Stemper et al. 2007; Venkatasubramanian et al. 2006; Chow and Zhang 2011). A better understanding of these changes requires a more in-depth examination, perhaps with the aid of better imaging techniques, which was not carried out during the experimental study. However, all the samples were stored and tested under similar conditions. Cylindrical sections of length 10 mm were cut out from the arteries and cut open along the longitudinal axis to form rectangular sections of approximately 5 mm width. In the present study, the following data from these experiments were used: multi-photon microscopy imaging in the initial no-load state, and the mechanical testing curves (uniaxial tests in in-plane circumferential, axial, and diagonal directions).

#### Image acquisition

**Fig. 2:**
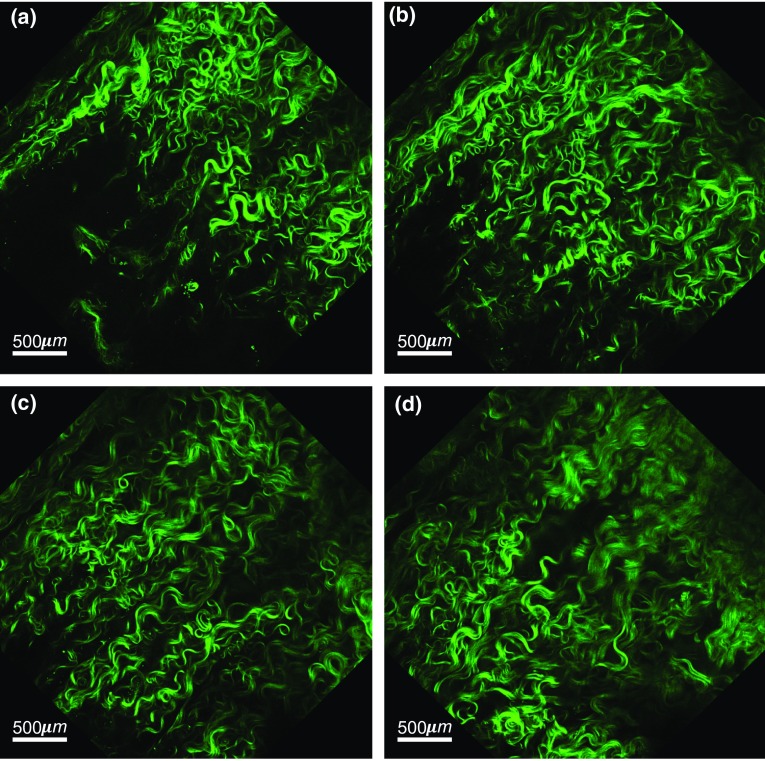
**a**–**d** Multi-photon microscopy images of adventitial collagen at depths of 25 $\upmu$m, 50 $\upmu$m, 75 $\upmu$m, and 100 $\upmu$m through the microstructure

Multi-photon microscopy images of the arterial specimens at zero load state were acquired with a Nikon A1R MP plus microscope. The excitation wavelength was set to 870 nm for optimal adjustment of auto-fluorescence and second-harmonic generation (SHG) signals. Microscopic images of the tissue were obtained, with the adventitial side facing the objective of the microscope (see Fig. 2). The images were acquired at an imaging resolution of 0.5 $\upmu$m in each direction with an imaging window of 512 $\times$ 512 $\upmu$m. The depth of the scan, which depended on the quality of the signal in response to the pulsed laser beam, varied in the range of 60–90 $\upmu$m. The total acquisition time for each stack with a scan speed of 2 frames per second, with two-frame averaging, took about 20–30 min.

#### Image processing

In order to develop a structurally accurate FE model of the adventitia, it is pivotal to study histology, to identify morphological parameters which define the structure, and to quantify those using reasonable stochastic formulations. Hence, the image stacks representing the collagen (SHG signal) were analyzed to extract relevant quantitative information regarding orientation, waviness, volume fraction, and diameter of this fibrous component.

The global orientation of a collagen fiber can be quantified with two parameters, polar angle $\phi$, which is the angle with respect to the axial–radial plane, and azimuthal angle $\theta$, which is the angle in the circumferential plane. Previous studies (Humphrey 2002; Wagenseil and Mecham 2009) showed that the orientation of collagen fibers in the axial–radial direction is negligible. Subsequently, we only focused on extracting the orientation of fibers in the axial–circumferential plane. The images were analyzed using an FFT analysis, which was previously utilized in studying the global orientation of collagen in the arterial wall (Polzer et al. 2013; Polzer and Gasser 2015). A total of 10 slices through a depth of 100 microns were analyzed and the measured azimuthal angles $\theta$ were averaged for all analyzed sections to obtain the orientation of collagen. The orientation distribution of fibers in the microstructure was then fitted using a finite mixture of von Mises distributions given by Eqs. 1 and 2, in order to later generate a numerical network by inverse random sampling.

1 $$\begin{aligned} f(\theta \Vert \mu _1, \mu _2, k_1, k_2, p)&= {p \frac{e^{k_1\cos 2(\theta - \mu _1)}}{\pi I_0(k_1)}} + {(1 - p) \frac{e^{k_2\cos 2(\theta - \mu _2)}}{\pi I_0(k_1)}} \end{aligned}$$

2 $$\begin{aligned} I_0(k)= & {} \frac{1}{\pi } \int _{0}^{\pi } e^{k\cos \theta } {\mathrm{d}}\theta \end{aligned}$$

where $k_1$ and $k_2$ are the concentration parameters defining the dispersion of each family of fibers, $\mu _1$ and $\mu _2$ are the mean orientation angles, and $0< p < 1$ is assigned to define the weight of each distribution. Finally, ${I_0 (k_1)}$ and ${I_0 (k_2)}$ are zero-order Bessel functions of type I given by Eq. 2, which act as a normalization parameter such that:

3 $$\begin{aligned} \int _{0}^{\pi } f(\theta \Vert \mu _1, \mu _2, k_1, k_2, p) {\mathrm{d}}\theta = 1 \end{aligned}$$

If *k* is small, the distribution tends to be uniform, whereas for very high values of *k* the distribution assumes a Dirac-delta function. As the value of *k* increases, the distribution approaches a Gaussian distribution with mean $\mu$ and variance $\frac{1}{k}$. Von Mises distribution is $\pi$-periodic such that $f(\theta ) = f(\theta + \pi )$. By varying the mean and concentration parameters, the above function can represent a wide range of collagen organizations, which can be used in modeling collagen networks in these tissues.

Collagen fiber waviness defines the initial crimp in the model and partially controls the strain at which collagen is recruited under loading, and therefore, it is significant in network reconstruction. For this reason, all the images of the stack were analyzed semi-automatically to obtain fiber characteristics such as end-to-end length $(l_{\mathrm{s}})$, and crimped length $(l_{\mathrm{c}})$. These parameters were measured using ImageJ tracing and measuring tools, which was also demonstrated by Rezakhaniha et al. (2012) using NeuronJ, an ImageJ plugin for neurite tracing and analysis. The waviness of a collagen fiber is then defined by Eq. 4.

4 $$\begin{aligned} W = \frac{l_{\mathrm{s}}}{l_{\mathrm{c}}} \end{aligned}$$

As the above equation suggests, *W* has a lower bound of 0 and an upper bound of 1. The higher the value of *W*, the straighter the fiber is. A value of 1 indicates that the fiber is not undulated. A beta distribution was used to characterize waviness distribution in the imaged sample. It consists of a continuous probability density function defined by Eq. 5, where $\alpha$ and $\beta$ are shape parameters, and $C(\alpha , \beta )$ is the normalizing constant. Maximum likelihood estimation was used to obtain the parameters of orientation and waviness distributions.

5 $$\begin{aligned} f(W, \alpha , \beta ) = C(\alpha , \beta ) {W^{\alpha -1}}{W^{\beta -1}} \end{aligned}$$

**Fig. 3:**
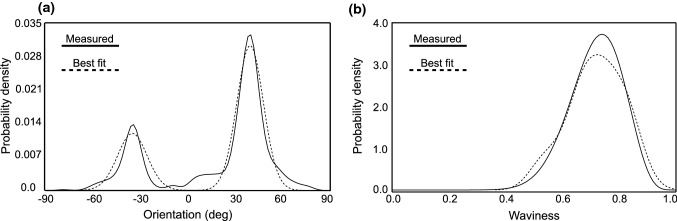
Measured versus identified probability density function for **a** orientation of collagen, **b** waviness of collagen

The result of using a finite mixture of von Mises distributions is illustrated in Fig. 3a. The resulting parameters from the identification process were $\mu _1$ = $-\,38.7{^{\circ }}$, $k_1 = 8.32$ and $\mu _2 = 40.7{^{\circ }}$, $k_2 = 10.03$, and $p = 0.31$. Similarly, the resulting probability density function for waviness with $\alpha = 15.78$ and $\beta = 6.21$ from the fitting process is illustrated in Fig. 3b.

To estimate the amount of collagen in the microstructure, a semi-automatic segmentation analysis was conducted in ScanIp^®^, a core image processing platform of the commercial software Simpleware^®^. As a first step, the images obtained from confocal microscopy are preprocessed in order to enhance the contrast of the fibers and diminish the background. The main segmentation step was realized with utilizing an array of pathfinding algorithms (shape detection, fast marching, geodesic active contours, etc). Seed points were defined manually by the user to track the fibers in the image stack following which the segmentation was achieved by solving a global optimization function specific to each algorithm used, which is based on the energy. Even though the segmented network could not be used to run a full-scale finite element analysis, it was used to approximately estimate the collagen content. An average value of 8 semi-automatic segmentations was taken as the representative volume fraction of collagen. This was done by taking the ratio of segmented volume to envelope volume, which yielded a volume fraction of $36.4 \pm 2.57$%. The obtained value of volume fraction is in accordance with the reported values in the literature (Chen et al. 2016, 2011).

#### Mechanical testing

**Fig. 4:**
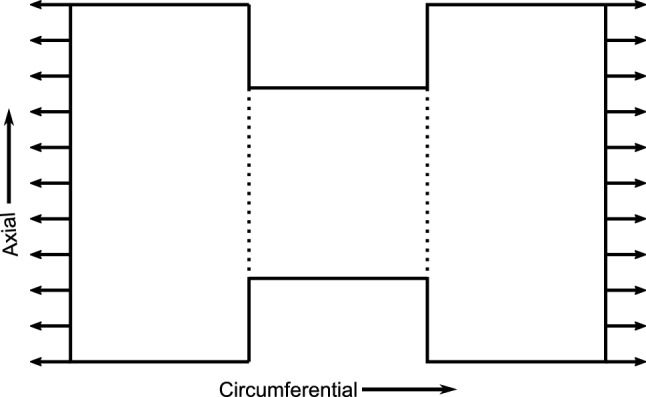
Schematic representation of the uniaxial tensile test sample

As mentioned in the above section, harvested arteries were used to perform uniaxial tension tests in three in-plane directions: circumferential, axial, and diagonal (Krasny et al. 2017) (Fig. 4). For this purpose, the samples were cut into a dog-bone shape. The cross-sectional area of the resulting strips was measured to be about $0.5 \pm 0.1\,{\mathrm{mm}}^{2}$. A high-precision tensile machine (Deben^®^ Microtest tensile/compression stage) with a load cell capacity of 150 N and a precision of 0.01 N was used for the uniaxial tensile tests. Before conducting the test, each sample was subjected to quasi-static triangular preconditioning. Following this step, the width of the tissue in the unloaded configuration was recorded four times and averaged. Uniaxial tension was then carried out at a controlled displacement until a target tensile force of 1 N was reached. The force measured at every loading state allowed the computation of first Piola–Kirchhoff stress $\sigma = \frac{F}{A_0}$, where *F* is the force at a given displacement and $A_0$ is the initial cross-sectional area. The stretch was measured based on the inter-clamp lengths measured at the initial state, which serves as the reference configuration, and at a given load (Krasny et al. 2017). For a more detailed understanding of the uncertainties arising from the experimental protocols, we direct the reader to Krasny et al. (2017). The obtained stress–stretch curves will be later used to identify model parameters and further validation.

### Mechanical model

#### Assumptions

The following assumptions were taken into account in building and analyzing the mechanics of the model:

1. Each fiber was assumed to be a sinusoidal curve; made up of segments of length $l_{\mathrm{seg}}$, whose direction of propagation is defined by a global orientation $\theta$ and local orientation $\theta _{\mathrm{rel}}$.
2. For each fiber, the global mean orientation was defined by the finite mixture of von Mises distributions given by Eq. 1.
3. The waviness of the fiber *W* was introduced by $\theta _{\mathrm{rel}}$ such that $W = \cos (\theta _{\mathrm{rel}})$.
4. The polar angle of the fibers, which is the angle out of the axial–circumferential plane, was assumed to be negligible for the reconstruction.
5. Every fiber was assumed to have its ends intersect the boundaries of the defined volume, such that no fiber lies dangling inside the volume. This assumption stems from the observations made on the microstructure of the adventitia under the confocal microscope of different arterial tissues. It was noted that the length scale of a collagen fiber is much larger than the field of view employed for this study, which is $500\,{\upmu }$m.
6. For global orientations of each sample, a similar probability density function with constant mean orientations and varying standard deviation was used to generate the network.
7. All the fibers were assumed to have the same diameter, instead of a distribution.
8. For the mechanics, only the collagen fiber network was simulated and analyzed. On the other hand, mechanical tests were performed on the whole tissue

#### Numerical network reconstruction

**Fig. 5:**
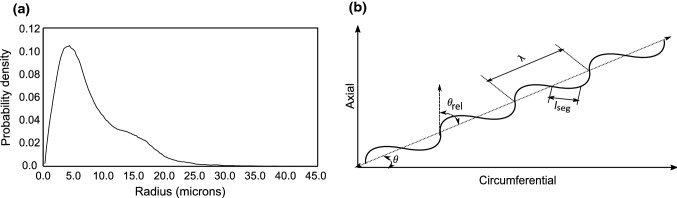
**a** Pore size radii distribution in the microstructure, **b** schematic representation of the reconstruction algorithm

Motivated by the work of Jin and Stanciulescu (2016) and Mauri et al. (2016), we defined the collagen fiber network as a discrete line network with each fiber represented as a worm-like chain, with a uniform diameter of 10 $\upmu$m (Carlisle et al. 2010; Sherman et al. 2015) for each fiber. This assumption was supported following a pore size analysis conducted on image stacks using a template matching algorithm as described by Krauss et al. (2012). The radius was taken to be the most probable value from the distribution of radius of fibers inside the tissue as shown in Fig. 5a. This enforced more support to the scale at which the mechanics of the collagen network are being studied. In this respect, a distinction can be made between the fibril and fiber scales based on the recorded diameter values (Ushiki 2002). It was also observed that at initial state some fibers tend to coalesce into bundles, which later separate upon application of load.

The steps followed in reconstructing the collagen network were as follows:

1. A 3-D volume of $500\,{\upmu }{\mathrm{m}} \times 500\,{\upmu }{\mathrm{m}} \times 100\,{\upmu }$m was specified to simulate the samples.
2. A uniform random distribution was used to specify the initial seed points of the fibers.
3. The orientation of each fiber $\theta \ \epsilon \ (0, \pi )$ was inverse sampled from its defined probability density function [Eq. (1)].
4. The waviness of the fiber W was inversely sampled from its defined probability density function [Eq. (4)].
5. The angle of each line segment relative to the fibers mean orientation, $\theta _{\mathrm{rel}}$, was computed based on the fiber waviness.
6. The fiber was propagated with segments of length $l_{\mathrm{seg}}$, which is the average wavelength measured from images, in opposite directions from the initial seed point until both ends meet the boundary of the volume.
7. Steps 2–6 were repeated until the desired fiber volume fraction was achieved.

A schematic of the above-mentioned fiber parameters is shown in Fig. 5b. The polar angle of the fiber was assumed to be insignificant and hence not considered in the reconstruction process. The parameter $\theta _{\mathrm{rel}}$ captures the waviness of fiber in the network. For sample no 4, the morphological parameters identified directly from the images were used as input, whereas for other samples an inverse approach based on the tensile stress–stretch response was used to identify modulus of collagen, axial connector stiffness, volume fraction, waviness, and orientation. This has been done for two reasons: the image stacks were only available for the first four samples, and the quality of the images for the other samples at no-distention state was adjudged to be insufficient to extract any meaningful morphological information.

#### Finite element model

The collagen fiber network obtained from the above reconstruction process was then used as an input for FE analysis. A custom script in Matlab^®^ was used to generate an input file to be imported to Abaqus^®^. It has been well documented that a pin-jointed random fiber network is stable for a coordination number of at least 4 at each joint (Picu 2011). As this is not the case with the generated network, which has a coordination number of 2 at each joint, the fiber segments were modeled using 3-D beam elements in the input file generation process. In order to simulate an equivalent truss-like behavior, the ratio of axial stiffness to bending stiffness for the beams was adjusted by reducing the radius from 5 to 0.1 $\upmu$m and increasing the elastic modulus proportionally, thus keeping the axial stiffness constant. The reconstructed network was meshed with 11000 B31H beam elements with the material behavior of each collagen fiber defined as incompressible and linearly elastic.

#### Boundary conditions

**Fig. 6:**
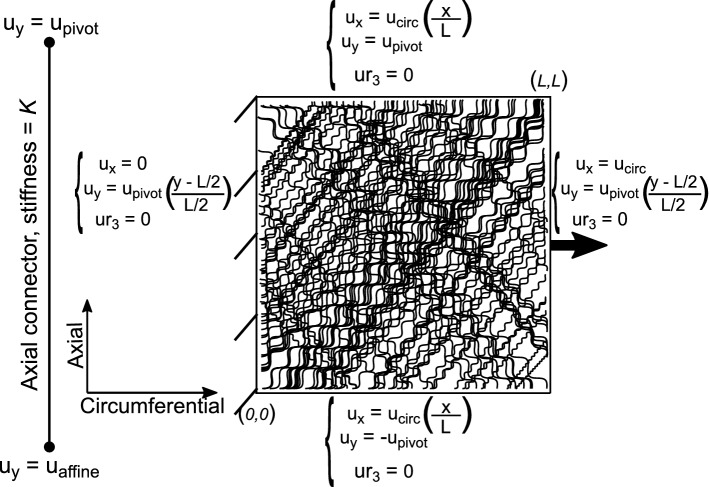
Boundary conditions used for the uniaxial tension simulations

The boundary conditions imposed on the model in order to simulate the uniaxial tension experiments are shown in Fig. 6. Node sets for each face where the fibers intersect with the boundaries were created. The desired axial stretch is achieved by displacing all the nodes on one axially symmetric surface, by a value $${u_{\mathrm{circ}}}$$, while restraining the other end. To induce the effect of apparent Poisson's ratio of the sample on the network, an axial connector was introduced and coupled to the transverse displacement degree of freedom of all boundary nodes. One node of the axial connector element was displaced in the traction direction according to the affine kinematics assumption while the other node (called pivot) was free (hence only transverse displacement possible). The stiffness of the axial connector was varied to control the displacement of the pivot. This pivot was then coupled with boundary nodes of the network to control their transverse displacement. Equation constraints were used to couple each node of the circumferentially symmetric boundary surfaces to the pivot as $$u_y= \pm$$$$u_{\mathrm{pivot}}$$. Similarly, a user-defined constraint was defined to couple each node on the axially symmetric boundary surfaces to the pivot as $$u_y= (\frac{y - \frac{L}{2}}{\frac{L}{2}})$$$$u_{\mathrm{pivot}}$$. The transverse deformation of the network was then controlled by the axial connector stiffness, a parameter of the present model.

Static analyses were performed using the Abaqus Standard solver. A displacement ${u_{\mathrm{circ}}}$ = 500 ${\upmu }$m (i.e., a stretch of 2) was applied in the traction direction. The main output of the simulations was the force versus stretch curve. The response was then used to compute the first Piola–Kirchhoff stress $\sigma =\frac{F}{A_0}$, where *F* is the tensile force computed as the sum of individual fiber reaction forces (projected along the traction direction) at the elongation boundary and $A_0$ is the initial cross-sectional area.

### Design of experiment approach for model evaluation

A reduced model of the previously presented model was constructed for two purposes. First, it was employed in an inverse identification approach to accelerate the identification of missing input parameters of samples exhibiting insufficient image quality. Second, the reduced model was used in a sensitivity analysis of the mechanical response to the main model parameters (see section below).

#### Input parameters

To build this reduced model, it was important to know the meaningful range of each parameter to be identified. All the parameters and their range of values used in constructing the reduced model are presented in Table 1. The modulus of collagen employed in this model was taken from previously reported experimental data (Aifantis et al. 2011; Dutov et al. 2016; Kato et al. 1989). About 20 simulations with varying stiffness values for the axial connector were run, following which the bounds of stiffness values for the axial connector element were chosen such that the lower bound value represented a free pivot and the upper bound value asymptotically approached affine kinematics behavior. A similar literature study helped in choosing the bounds for collagen content in the adventitia and initial collagen waviness. Following the choice of bounds for each input parameter, a uniform sampling method was employed to sample input points in all dimensions of the parameter space. The principle of this technique is to partition space of m parameters into $\prod _{i=1}^{m} n_i$ parts, where $n_i$ is the number of intervals the *i*th input parameter range is divided into. Then, $\prod _{i=1}^{m} n_i$ sets of m dimensions were created such that each set contains exactly one partition of each parameter.

A total of 7200 simulations were performed for extracting the uniaxial response and microscale mechanics of networks generated by varying the parameters presented in Table 1. For the purpose of avoiding over-complexification, the mean fiber orientations and weight of individual distributions were not varied from the values extracted from the image stacks. Only the standard deviation was varied but assumed to be the same for both families. The standard deviation for a given von Mises distribution can be computed using the equation $\sigma ^{2} = 1 - \frac{I_1(k)}{I_0(k)}$, where *k* is the concentration parameter as described in Sect. 2.1.2. This allowed reducing the number of parameters to be varied for orientation distribution to one in place of five and yet be able to simulate a wide range of initial network configurations. The resulting stress–stretch data from each simulation were fed to a six-dimensional gridded interpolant in Matlab^®^. The first dimension of the grid corresponded to a macroscopic stretch value, while the other five dimensions correspond to the input parameters. This helps in extracting the stress–stretch response of any configuration of parameters defined within the bounds of the input parameters.

**Table 1:** Range of input parameters

Parameter	Lower bound	Upper bound
Parameter list		
Collagen modulus	60 MPa	300 MPa
Axial connector stiffness	0.05 Nm$^{-1}$	20 Nm$^{-1}$
Volume fraction	20%	40%
Waviness ($\mu$)	0.67	0.83
Orientation ($\sigma$)	$2.5{^{\circ }}$	$10{^{\circ }}$

#### Responses

The overall response of the microstructure depends on the parameters used to construct the model, which are shown in Table 1. In order to study the influence of varying these model parameters on the global response of the microstructure, two independent responses of the model were specifically chosen: the final slope, and the recruitment stretch of the stress–stretch curve. The final slope response was chosen such that it is indicative of the macroscopic stress of the network. This implies that higher the final slope of the network, higher will be the global stiffness of the network. Thus, the index is helpful in identifying the various possible combinations of constitutive and morphological parameters which result in stresses leading up to rupture. On the other hand, collagen recruitment is a stiffening mechanism observed in the arterial wall and other collagenous tissues and marks the onset of collagen being the principle load-bearing component. The recruitment stretch response was chosen in order to quantify the mechanical behavior of collagen in the network. There is not a lot of information in the literature as to what factors influence the recruitment of collagen. The commonly accepted notion is that the waviness of the fibers controls collagen recruitment (Hansen et al. 2002). Thus, the evaluation of this response is used to quantify the effect of other parameters on collagen recruitment.

*Final slope:* The final slope was computed as the slope of the stress–stretch curve comprising the final 10% of the stretch. This index, unit of MPa, was computed for all the 7200 simulations, and the result was stored as a vector $s_{final} = \{s_1, s_2, \ldots , s_{7200}\}$.

*Recruitment stretch:* The recruitment stretch of a collagen network can be defined as the global stretch at which collagen starts to act as the major load-bearing component. The recruitment stretch was computed, in this study, as the global stretch at which the rate of change of slope was found to be the highest in the stress–stretch curve. The index for recruitment stretch is unitless. Like for the final slope, the index was computed for all the 7200 simulations and the result was stored as a vector $\lambda _{\mathrm{recruitment}} = \{\lambda _1, \lambda _2, \ldots , \lambda _{7200}\}.$

#### Linear and nonlinear regression for sensitivity analysis

Five-dimensional response surfaces $s_f$ and $\epsilon _r,$ respectively, represent the final slope and recruitment stretch indices as a function of the five-dimensional input parameter matrix (*X*) described by Eq. 6:

6 $$\begin{aligned} \begin{aligned} s_{\mathrm{final}} = s_f\{X\} + c_s \\ \lambda _{\mathrm{recruitment}} = \lambda _r\{X\} + c_{\lambda } \end{aligned} \end{aligned}$$

where $c_s$ and $c_{\epsilon }$ are the residuals obtained during the fitting process. Each of the response surfaces was fitted using regression analyses, which were performed in Matlab^®^ using the in-built regression models. First, a linear regression with 5 linear terms and 1 constant term was fitted to study the global influence of each input parameter on each of the chosen indices. Then, a quadratic regression with 5 linear terms, 14 quadratic terms and 1 constant term was fitted to study the interactions between input parameters for each response. Practically, each of the regression analyses utilized the whole parameter space of the reduced model. Since all parameters have different units, they were normalized on a scale of 0 to 1, where 0 corresponds to the lower bound and 1 corresponds to the upper bound of the parameter.

In order to assess the accuracy of the fitted models, two different statistical methods were used. The root mean squared error (RMSE) value and the *R*-squared value were calculated for both linear and quadratic regressions. Finally, in order to assess the meaningfulness of each predicted regression coefficient, the *p* value was used. Using this information, regression coefficients with an insignificant *p* value (*p* value $>\,0.05)$ were neglected and the regression analyses were performed again.

### Identification of model parameters

The 6-D gridded interpolation function was used as a reduced model in order to speed up the identification process of the missing parameters including collagen modulus, axial connector stiffness, volume fraction, waviness, and orientation for each sample.

#### Validation on sample no 4

Through a morphological analysis, we were able to extract the morphological parameters required to build the model for sample no 4, hence leaving us with only collagen modulus and axial connector stiffness to be identified. Using the experimental stress–stretch data, it was possible to uniquely identify these two parameters which optimize the predictive capability of the model to represent the measured behavior of the microstructure. To do so, the geometry of the microstructure of sample no 4 was built following the algorithm described in Sect. 2.2.2; and a genetic algorithm (MathWorks, 2015), which takes into account the initial population data for the gridded interpolants, and their input parameters as specified in Table 1 was used in Matlab^®^ to perform a constrained optimization. An *R*-squared function given by the equation below was used as the objective function to minimize the error between experimental and model force vs. stretch curves. In the equation below, *n* represents the number of data points where stress–stretch data were collected from the experiments. Corresponding values of stress–stretch data were computed from the gridded interpolant in order to complete the equation below. The *R*-squared function was defined in a negative sense owing to the global minimization procedure adapted in the genetic algorithm. The constraints for the above function were given in the form of bounds for the input parameters. For the morphology parameters, the values were fixed to the extracted data, whereas for the constitutive parameters, bounds were assigned as defined in Table 1. A sufficient number of independent runs were performed in order to obtain a global minimum. Finally, once an optimal set of constitutive parameters was obtained, the model was compared with experimental data for validation.

7 $$\begin{aligned} \begin{aligned} R{\mathrm{-Squared}} = \frac{\sum \nolimits _{i=1}^n ({\mathrm{Experiment}}(i) - {\mathrm{Interpolant}}(i))^{2}}{{\sum \nolimits _{i=1}^n ({\mathrm{Experiment}}(i) - {\mathrm{mean}}({\mathrm{Experiment}}))^{2}}} - 1\\ \end{aligned} \end{aligned}$$

#### Identification of model parameters on other samples

Unlike for sample no 4, for the other 5 samples the complete input parameter set (i.e., morphological + constitutive) was inversely identified from experimental data. As described earlier, a genetic algorithm with the objective function resembling an *R*-squared function was used to perform the constrained optimization. In this case, the constraints were applied in the form of bounds for all parameters as defined in Table 1. A series of independent runs were performed to ensure a global minimization.

## Results

In order to demonstrate the predictive capability of the proposed model, the computational results obtained from the simulations were compared to experimental data of six samples of rabbit carotid arteries under uniaxial tension [previously published in Krasny et al. (2017)]. It is interesting to note that the uniaxial tension tests were performed on the whole tissue, while the reconstructed microstructure represents only the adventitial layer. This was done due to the fact that the depth to which confocal microscopy images could be obtained with reasonable quality was limited. This often times resulted in image stacks representing a thickness of less than $200\,\upmu$m, while the tissue is much thicker than that. To overcome this limitation, comparisons were made on stress data, assuming homogeneous stress through the sample thickness. Also, it is interesting to note that comparisons were made beyond a high stretch value, where collagen is usually considered to be the principle load-bearing component.

### Model validation

**Fig. 7:**
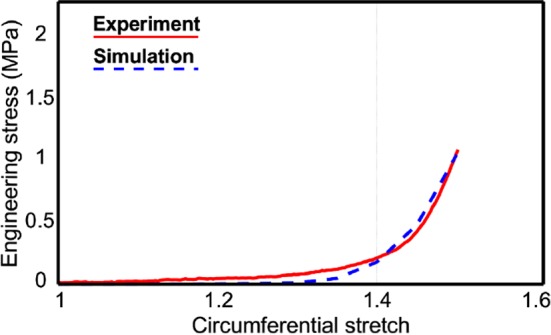
For sample no 4 comparison of stress–stretch results from finite element simulations with experimental data

First, we present the model parameters corresponding to sample no 4 in Table 2, for which only the modulus of collagen and axial connector stiffness were identified. The comparison between experimental data and the numerical response of the model is presented in Fig. 7a. Note that since we are interested in the mechanical response of the collagen fiber network as opposed to the whole tissue, the relevance of comparison mainly lies in the recruitment stretch value and beyond that point, where the simulation results demonstrated an accurate prediction of the mechanical response. In this case, an *R*-squared value of 0.968 was obtained, which confirmed the fitting accuracy.

**Table 2:** Optimal parameter set for sample no 4

Collagen modulus	Axial connector stiffness	Volume fraction	Waviness	Orientation ($\sigma$)
297 MPa	0.955 Nm$^{-1}$	37.1%	0.80	$3.55{^{\circ }}$

### Parameter identification from experimental data

**Fig. 8:**
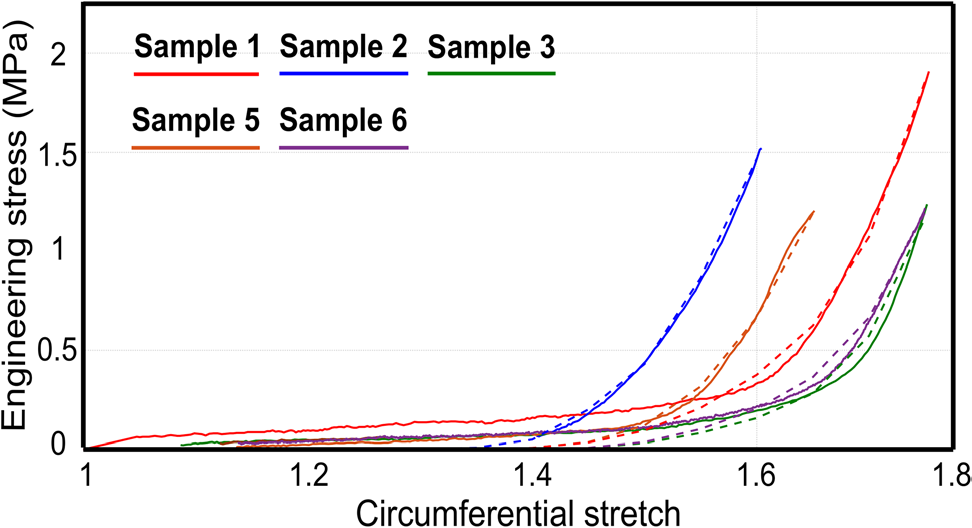
Comparison of results obtained from finite element simulations with experimental data of different arterial samples

The methodology described above was applied to 5 other samples previously tested experimentally to obtain their optimum input parameter set. The parameters obtained from multiple runs of the genetic algorithm for each sample are presented in Table 3. It can be seen that all the parameters lie in their respective range without reaching range limits. The comparison between experimental data and the simulation results for these samples is shown in Fig. 8. In each case, an *R*-squared value above 0.9 was obtained, with a minimum obtained value of 0.92 for no 3. Note that the insignificant contribution of fibers to the global stress until the point of recruitment can be explained by the uncrimping process they undergo as the sample stretches. This aspect is addressed later in Sect. 3.4.

**Table 3:** Optimal parameter set for samples 1, 2, 3, 5, and 6

Sample	Collagen modulus (MPa)	Axial connector stiffness (Nm$^{-1}$)	Volume fraction (%)	Waviness	Std deviation of orientation
1	163.34	1.78	29.03	0.757	5.21°
2	283.86	3.81	28.53	0.793	4.33°
3	80.64	1.06	31.98	0.746	5.86°
5	194.6	4.4	36.80	0.769	3.98°
6	70.45	1.39	29.35	0.757	6.16°

### Sensitivity analysis

#### Linear model

A linear regression model with 6 terms was identified to study the linear effect of each parameter on the overall response. The linear regression model with 6 terms exhibited an RMSE value of 0.246 MPa, an *R*-squared value of 0.94 for final slope and an RMSE value of 0.0328, an *R*-squared value of 0.92 for recruitment stretch. The computed sensitivity coefficients for each output are presented in Fig. 8. The value of a coefficient signifies the amount of influence the corresponding parameter has on the response. For instance, in evaluating the sensitivity on the final slope, it was observed that modulus of collagen has the most influence. By increasing its value on the normalized scale from 0 to 1 (i.e., 60–300 MPa), the final slope of the response increases by 7.15 MPa. On the other hand, the initial waviness in the network exhibited the most negative influence on the final slope of the response. A sensitivity coefficient of − 1.92 MPa signifies that by increasing the initial waviness in the network from 0 to 1 (i.e., 1.2–1.45 straightness), the final slope reduces 1.92 MPa. The most influential parameter for recruitment of collagen was the initial waviness in collagen. By increasing its value from 0 to 1 on the normalized scale, stretch at which collagen is supposed to be recruited increased by 0.19. While collagen modulus, volume fraction, and orientation variance had almost no influence on collagen recruitment, axial connector stiffness displayed a slight negative influence with a sensitivity coefficient of − 0.028.

**Fig. 9:**
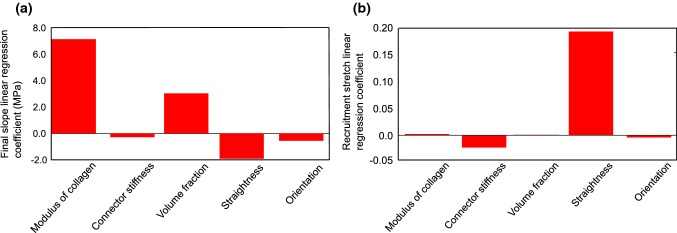
Sensitivity indices from linear regression for **a** final slope response, **b** recruitment stretch response

#### Nonlinear model

A nonlinear regression model with 20 terms was also built to analyze the nonlinear effects and interactions between parameters. An initial analysis revealed some parameters which did not have any significance on the output (*p* value $\,> 0.05)$. Following this, the analysis was repeated with 18 terms for final slope and 13 terms for recruitment stretch. The nonlinear regression model with 6 terms exhibited an RMSE value of 0.0889 MPa, an *R*-squared value of 0.992 for final slope and an RMSE value of 0.312, an *R*-squared value of 0.902 for recruitment stretch. The nonlinear contribution of individual parameters and their interactions are presented in Fig. 9, from which they can be interpreted according to the intensity of the color plot. The coefficient values which were found to be insignificant (*p* value $\,> 0.05)$ are displayed in black. The quadratic terms corresponding to each parameter revealed that none of them exhibit a strong nonlinear response w.r.t. both final slope and recruitment stretch. Waviness exhibited the most nonlinear behavior in both cases. For the final slope response, modulus of collagen with volume fraction, and modulus of collagen with waviness exhibited significant interactions. The interaction between modulus of collagen and waviness was found to be negative, which is reasonable as they have a reciprocal influence on it. For the recruitment stretch, the interaction between collagen modulus and every other parameter was found to have very little significance, whereas axial connector stiffness and waviness showed the most interaction.

**Fig. 10:**
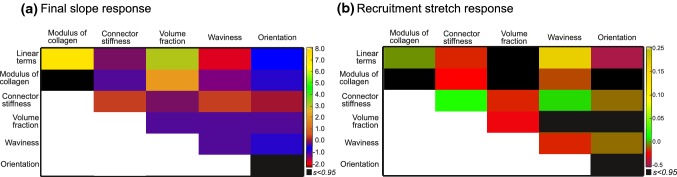
Sensitivity indices from nonlinear regression for **a** final slope response, **b** recruitment stretch response

### Morphological changes of the fiber network

#### Reorientation

**Fig. 11:**
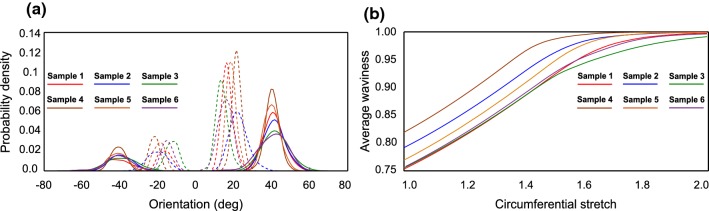
**a** Comparison of orientation distribution of collagen at initial state in each sample to each sample’s maximum stretch state, **b** Evolution of average waviness of the collagen network in all samples

The mechanics of the model collagen network is completely determined by the constitutive behavior of the fibers, their waviness and orientation, and the network density. It has been well documented in the experimental literature that collagen fibers gradually reorient toward the load direction (Niestrawska et al. 2016; Krasny et al. 2017). To understand this behavior, the evolution of the orientation distribution of fibers was studied at various stages of loading. The orientation distribution of collagen in its initial configuration and at a maximum stretch of each sample is presented in Fig. 10a. The analysis of fiber angles in their final configuration suggested that fibers preferred orienting themselves close to the traction direction. The dispersion of fibers was also observed to reduce as fibers tend to align toward the loading direction. The initially preferred orientations of collagen fibers with respect to the circumferential direction in all samples were approximately $40^{\circ }$ and $-\,40^{\circ }$. As the samples were stretched uniaxially in the circumferential direction, the preferred orientations changed to approximately $18^{\circ }$ and $-\,18^{\circ },$ respectively. The amount of reorientation in each sample was dictated by its transverse stiffness and global stretch. This was computed as the average of percentage reorientation of all fibers in a sample. At a given global stretch, the sample identified with least axial connector stiffness underwent most reorientation. For instance, at a global stretch of 2.0 in the simulations; sample no 4 underwent the maximum reorientation of about 85.63% followed by sample no 3 with 85.14%, sample no 6 with 77.89%, sample no 1 with 72.81%, sample no 2 with 72.05%, and sample no 5 with 70.62%. Nevertheless, at their respective maximum global stretches of 1.75 and 1.52 in the experiments, sample no 3 underwent a maximum reorientation of about 67.45%, and sample no 4 a reorientation of about 46.33%, signifying that the axial connector stiffness bore lesser influence on the amount of reorientation as compared to the global stretch (Fig. 11).

#### Straightening

Collagen fibers which were undulated in their initial state underwent a straightening process as the load increased. It can be seen from Fig. 10b that the average waviness in the network almost reaches 1 at high stretches. It was observed that, for some samples, even at high loads the collagen fibers remained slightly crimped. This might be due to a high initial average waviness combined with the amount of transverse displacement induced by the pivot governing transverse response. For two samples with approximately equal initial waviness, the amount of reorientation had a direct impact on the final average waviness in the model. For instance, sample no 3 and sample no 6 had identical initial waviness of about 0.752, which resulted in 0.984 and 0.996, respectively. In the above scenario, sample no 3 had to undergo a reorientation of 85.14%, whereas sample no 6 underwent a reorientation of about 77.89% signifying that the straightening process for a fiber undergoing higher reorientation is slower than for a fiber undergoing lesser reorientation.

#### Non-affine behavior

**Fig. 12:**
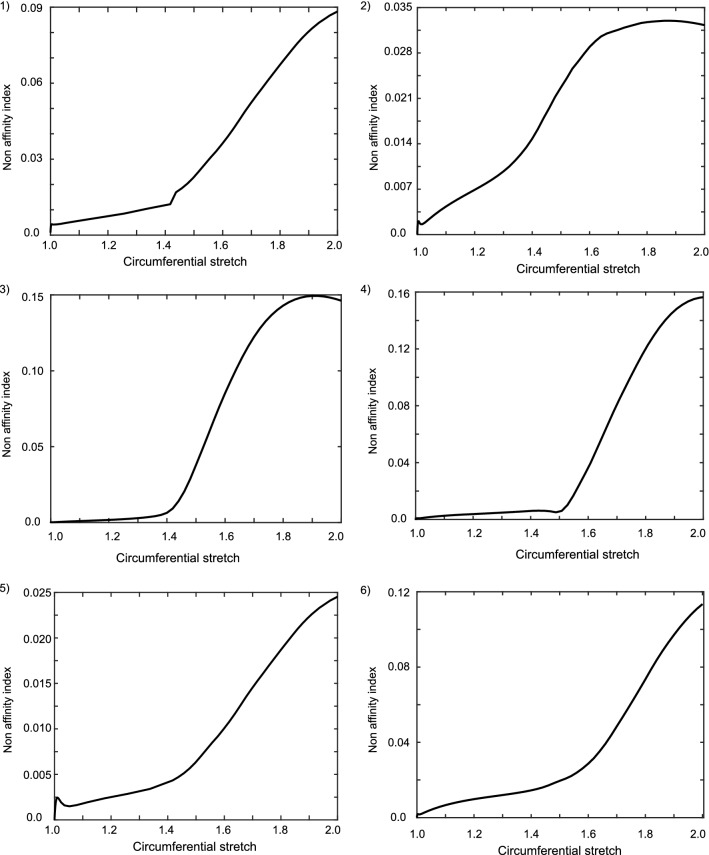
Evolution of non-affinity index with stretch computed for all samples

The morphological rearrangement of collagen also determines the apparent non-affine nature of the network deformation. A random fiber network with low network density and a high ratio of axial to bending stiffness is more compliant with non-affine behavior (Picu 2011). To this effect, the computed collagen fiber displacements were used to study the apparent non-affinity in the network using a strain-based metric defined in Eq. 8.

8 $$\begin{aligned} NA(r) = {||(E - E_{\mathrm{affine}})^{2}||} \end{aligned}$$

where $E = {\epsilon _{{11}}, \epsilon _{{22}}, \epsilon _{{12}}, \omega _{{12}}}$ is a vector containing the strain and rotation components, $E_{\mathrm{affine}}$ is the corresponding affine vector. The length scale at which these strain values were probed is defined by *r*, which was chosen to be $l_{\mathrm{seg}}$, the distance between the cross-links. To understand the evolution of non-affine behavior of these networks, the obtained non-affinity value was plotted against far-field stretch and is reported in Fig. 12. The amount of non-affinity computed using the above method showed a clear tendency that samples with low transverse stiffness exhibited a higher non-affine behavior as compared to samples with high transverse stiffness. This can be observed to be the case from the figure where sample 4 exhibited the highest non-affinity of almost 16% and sample 5 exhibited the lowest of about 2.5%. The reason for such a drastic difference between the highest and lowest is that for sample 5 the identified transverse stiffness corresponded to a near affine behavior. The amount of non-affinity in the network can be quantitatively analyzed at various stages of loading, which revealed a similar evolution trend in all samples. The initial phase was characterized by low non-affine values with a very gradual increase when increasing global stretch. Beyond this, the non-affinity increased considerably when increasing global stretch. The point of separation between these two phases was observed to be the recruitment stretch of collagen.

### Analysis of the micro–macro-relationship

The model was further used to analyze the micro-level mechanical state of each sample at stretches corresponding to average peak wall stress at rupture. This kind of analysis facilitates the investigation of physical quantities that are not (yet) accessible through experiments. A value of 1 MPa, representative of peak wall stress at rupture, was taken from literature (Gasser et al. 2010; Fillinger et al. 2002), and a simulation was run again for each model in order in to reach that stress value and study the corresponding mechanical state of the fiber network.

**Fig. 13:**
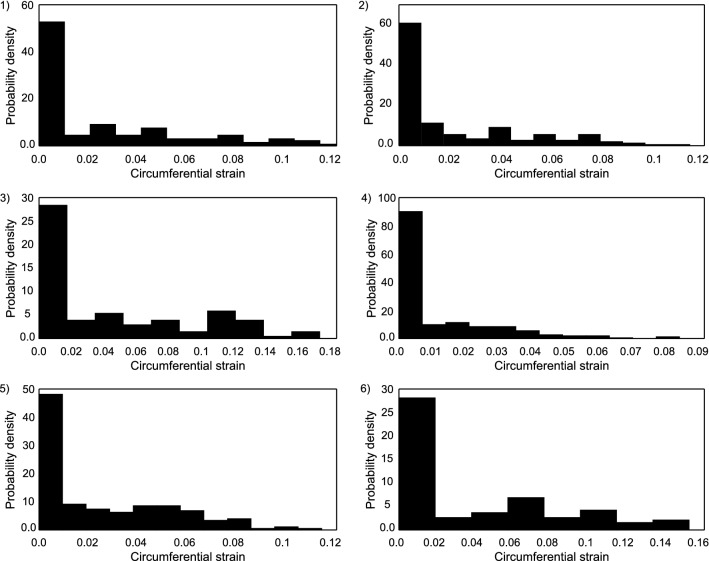
(1–6) Distribution of axial strain in collagen fibers in each sample computed at a global stress of 1 MPa

The microscopic strain distribution in fibers for each sample at this macroscopic stress of 1 MPa is shown in Fig. 13. It is possible to observe from the histograms that majority of the fibers in each sample underwent an axial strain of less than 5%. Samples 1, 3, and 6 exhibited axial strains of more than 10% in several fibers. It is possibly a result of high macroscopic stretch required for each of these samples to achieve stress of 1 MPa. It should also be observed that these three samples were identified with relatively lower values of collagen modulus and higher values of initial collagen waviness. From the sensitivity analysis conducted, it was found that these two parameters bear the most influence on the final slope response. Samples 2, 4, and 5, which were identified with high collagen modulus and low initial waviness, had all fibers with an axial stretch less than 10%. Thus, it can be concluded that axial strain in individual collagen fibers at a given macroscopic stretch depends indirectly on their elastic modulus and initial crimp.

**Fig. 14:**
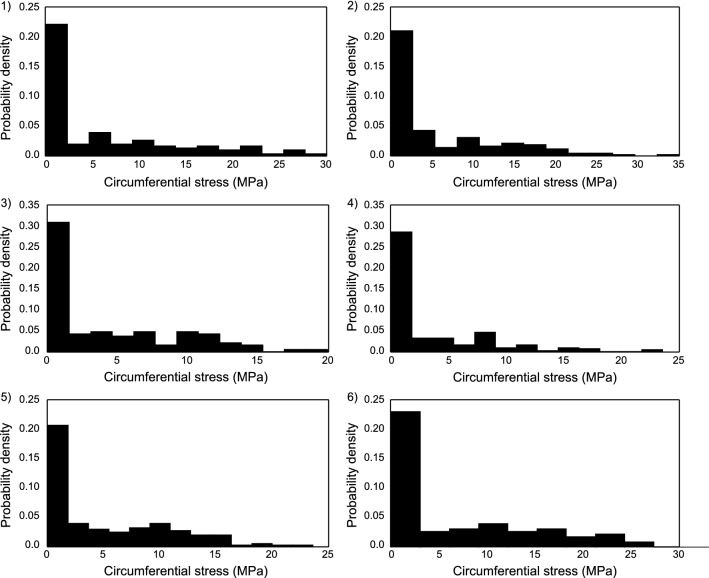
(1–6) Distribution of axial stress in collagen fibers in each sample computed at a global stress of 1 MPa

A similar analysis of the microscopic stress distribution of fibers at macroscopic stress of 1 MPa is shown in Fig. 14. It can be noticed that even though samples 3 and 6 have been identified with a relatively low elastic modulus, the fibers exhibit high-stress values. This is possibly due to the fact that they exhibit high axial strains in fibers as noted in Fig. 13. Although it might be imperative to conclude interesting remarks about fiber rupture based on these stress values, the heterogeneity in the material constitutive parameters should be noted. There is not enough data in the literature to facilitate such an analysis. Hence, only strain distributions were used as will be described in Sect. 4.5.

## Discussion

### Main contributions

In this paper, a discrete random network model of the tunica adventitia built from experimental data is presented, to capture the mechanical behavior at the scale of the fibrous microstructure. Computing and analyzing the mechanical phenomena occurring at the scale of the microstructure has been a focused area in arterial mechanics. Histomechanical constitutive models are used widely to distribute macroscopic stress to various microstructural components and correlate macroscopic loading to microscopic component responses. Previous attempts at developing an anatomically representative finite element model for arterial wall layers did not investigate the morphology but rather relied on inversely identifying the morphological parameters. With the method specified here, morphological analysis was made to study the distribution of collagen fiber orientations, waviness, diameter and their volume fraction in the tissue. The proposed model is anatomically based, as it incorporates this information by employing an inverse random sampling approach. Recently, it has been realized that macroscale rupture occurs at locations of localized strain concentration, which interestingly was in contradiction to the notion that rupture occurs at the location of maximum stress (Romo et al. 2014). While it is possible to investigate the mechanical behavior of individual arterial wall layers with analytical models, it is interesting to develop a microscale finite element model of the adventitia to study microscopic mechanical phenomena responsible for the risk of rupture. The main objective of this work was to introduce a method that can be readily implemented with necessary image processing and FE analysis tools.

The histology-based reconstruction presented in this paper is general in the sense that it can be applicable to many stochastic fibrous microstructures. The orientation of fibers is one of the two important parameters required for the reconstruction. The presence of two distinct families of fibers was evident from the microscopy images. Hence, the orientation distribution of collagen in the network obtained through an FFT analysis was later fitted to a mixture of von Mises distributions (with each distribution representing a family of fibers). Also, it was observed from the analysis of the images that the number of fibers in each family was not the same as it was normally believed to be. To this effect, a weighting parameter *p* was introduced to take into account the contribution of each family of fibers. The model also takes initial waviness of collagen into consideration which was fitted to a beta probability distribution. The initial waviness of collagen coupled with dispersed orientations controls the highly nonlinear mechanical response of the network. To reconstruct a numerical network which represents the actual collagen network as accurately as possible, the diameter and content of collagen were also extracted from the image stacks, which had not been previously considered. The entire workflow of this forward approach of reconstructing numerical network from morphology data was conducted on a sample no 4.

### Comparison with the literature

To ensure fast applicability of the method on a wide range of experimental data from other samples, a reduced model was constructed using constitutive and morphological parameters as inputs. Constrained optimization was then conducted to inversely identify the parameters of all samples from which obtaining morphological data was not feasible due to image quality. The data from uniaxial tensile tests were used in that case. All the identified parameters (see Table 2) fell in an acceptable range of values reported in the literature (Rezakhaniha et al. 2012; Chen et al. 2011; Sherman et al. 2015; Chandran 2005; Schriefl et al. 2012). Since we were mainly interested in studying the behavior of collagen in the adventitia, the response of the network prior to the point of collagen recruitment was not taken into consideration. The capability of this modeling method was demonstrated by comparing the numerical simulation result in experimental data (Figs. 7, 8). The satisfying agreement with experimental data demonstrated the capability of the model to predict the macroscopic response of the tissue at high strains for a wide range of microstructural arrangements.

### Sensitivity analysis

Following the comparison of numerical results with experimental data, a comprehensive parameter sensitivity analysis was conducted to establish meaningful relationships between individual model parameters and macroscopic mechanical response determinants. The responses studied include the stretch at which collagen is recruited and the stiffness of the tissue following collagen recruitment. Linear regression model for the final slope response revealed that axial connector stiffness and orientation dispersion had negligible impact compared to those of collagen modulus, waviness and volume fraction. As previously shown in Gizzi et al. (2014), Chen and Kassab (2016), it was observed here that collagen waviness had a negative influence on the final slope response. Regarding the recruitment stretch, collagen waviness was found to be the main influencing parameter. This aspect was also briefly studied from 3-D rendered MPM images (Hill et al. 2012), where it was found to bear a direct relationship. This finding is in line with the present linear regression coefficient predicting an increase in recruitment stretch with an increase in collagen waviness. A comparative study conducted by Holzapfel and Ogden (2017) showed that collagen fiber dispersion effects the macroscale mechanical response significantly. However, it is to be noted that the range of dispersions considered in their study is much higher than what we explore with our model. In any case, a quantitative comparison of the explored sensitivity indices could not be made at this moment. Although the linear regression helped to understand the overall effect each parameter exhibited on each response, the nonlinear regression model was found to be a more accurate representation in both cases with lesser RMSE and higher *R*-squared values. However, it did not highlight any remarkable combined effect.

### Fiber kinematics

**Fig. 15:**
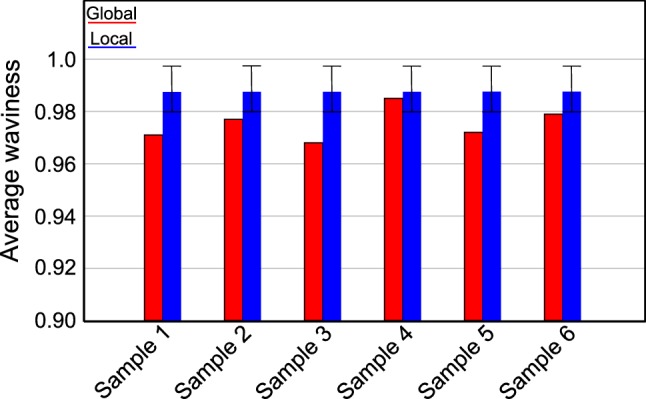
Comparison of computed recruitment stretch for collagen in each sample to theoretical estimates

The kinematical reorganization of collagen fibers under load was emulated by the model as shown in Fig. 10 and is in coherence with previous investigations. The amount of transverse deformation combined with the increasing tension the fiber network has to bear dictated the amount of reorientation in collagen fibers. A review on the mechanics of random networks of various densities in Picu (2011) suggested that collagen fiber reorientation is explained by non-affine kinematics. We always observed that collagen reorientation predicted by the model was greater than the affine prediction. It was also computed that the average error in reorientation prediction made by an affine assumption was 22.65% ± 6.02%, which is in agreement with the average value of 25% reported from experimental investigation (Krasny et al. 2017). As the load increased, collagen fibers also underwent a straightening process which combined with reorientation to control their recruitment. It was observed that the average waviness in the network did not reach 1 meaning not all fibers were straightened at the end of loading. To understand the recruitment phenomena, a mathematical foundation for fibril recruitment in tendons was developed by Bevan et al. (2018), based on which they estimated average critical recruitment waviness in arteries as 0.996. For further analysis, for each sample, collagen recruitment stretch was computed as the global stretch at which the rate of curvature change was found to be the highest. As can be seen from Fig. 15, recruitment of collagen is in a comparable range of the average experimental value. It was also noted that the rate of straightening reduced significantly beyond the point of recruitment stretch. This result confirms that the macroscopic notion of recruitment stretch defined from macroscopic stretch–strain curves (Bevan et al. 2018; Hill et al. 2012) is closely related to the microscopic, fiber-level, recruitment stretch which could be investigated thanks to the present model.

### Rupture prediction

**Fig. 16:**
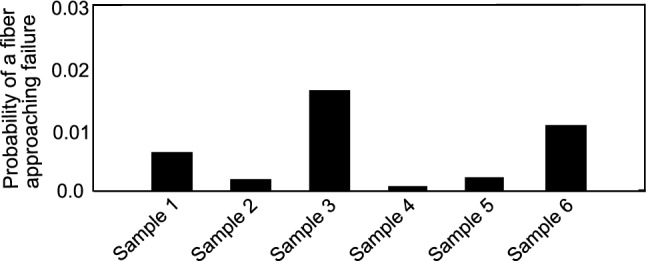
Bar chart of computed values of strain-based failure probability of collagen in all the samples

The study of the macroscopic mechanical response of arterial wall layers is well documented in the literature with the use of phenomenological and structural constitutive models (Chuong and Fung 1983; Raghavan et al. 2000). While these models accurately predict the mechanical behavior of the tissue, they do not provide any insight into the phenomena occurring at the microstructure level. To this effect, the proposed model was used to investigate the microscopic strain and stress distributions in individual fibers constituting the network. This was used to study the mechanical state of collagen fibers at a macroscopic stress level corresponding to rupture. Interestingly, this analysis indicated that fibers approaching their tensile limit were closely oriented toward the circumferential direction in comparison with their mean orientation at that loading level. Following that, for each sample, the strain distributions at load corresponding to rupture were analyzed and fitted to a probability density distribution. This could be used to compute the probability of finding a fiber in any given range of tensile stress/strain, and hence estimating a rupture probability index. Indeed, it was noted that for some samples a small percentage of fibers approached their tensile limit. Their probability is quantified and presented in Fig. 16. It was noted that for a sample no 3, which was subjected to maximum stretch the probability was found to be the highest. From this, we can hypothesize that damage initiation would possibly occur at a local level before propagating toward macroscopic rupture. By utilizing this information, the model could serve as a probabilistic tool for predicting rupture based on the morphological information of the microstructure. As current experimental data do not include the necessary information for quantifying rupture, one of the main perspectives of the present study would be to gather experimental data at loading conditions reaching rupture. These data would be then fed to the model in order to quantify meaningful rupture criteria based on structure–function relationships.

### Limitations

The model presented in the study is not without limitations, which can be discussed as below:

1. We recall, first, that the study was limited to the mechanics of collagen network in the adventitia as the effects of the connective tissue and other microstructural components were not considered. This choice lied onto the common hypothesis that at high loads beyond physiological pressure levels, collagen acts as the principal load-bearing component in the arterial wall.
2. Physical interactions between the fibers and the ground matrix were not considered in the current model. This was rather modeled phenomenologically using a connector element which is kinematically linked to the boundary edges of the model, thus controlling the overall non-affine behavior of the network and enabling compressibility in the collagen network.
3. Interactions between individual fibers are not considered in this model assuming that their effect would be of a lesser order compared to tension effect in the overall response to uniaxial tension.
4. In the presented model, collagen fibers were assumed to be oriented mostly in the axial–circumferential plane. Hence, the orientation distribution obtained from image stacks only corresponded to the azimuthal angle, neglecting the effect of a polar angle like in many studies in the literature (Jin and Stanciulescu 2016; Holzapfel et al. 2015; Gasser et al. 2006). For a more realistic representation of the microstructure, 3-D segmentation tools which can accurately render the fiber morphology are required. At this moment, the quality of the images prevents such analysis which would be beyond the scope of this study.
5. We also assumed the same collagen orientation density distribution for all samples. Nonetheless, it can be seen from the reported data in Krasny et al. (2017) that the assumed distribution is within the limits of average orientation density of the measured samples. This is a strong simplification, however; but when it is possible to experimentally measure this distribution, it can easily be introduced in the model.
6. Next, the experimental image stacks corresponded to a depth of 100 ${\upmu }$m owing to the quality of the signal at in-depth focal positions. Therefore, morphological information extracted from the image stacks was assumed to be representative of the tissue, which may be questioned.
7. Each fiber in the network was modeled as a chain of segments alternating around a given orientation. This led us to the assumption that all fibers had their ends on the boundaries of the modeled volume. Although this did not compromise the overall stochastic nature of the network, it is worth noting that realistically, fibers which dangle inside the microstructure could possibly exist.
8. Finally, the material parameters identified for the samples were only based on uniaxial tension loading data. For other loading scenarios, the applicability of these identified parameters has not yet been verified and will be the purpose of future investigations.

**Fig. 17:**
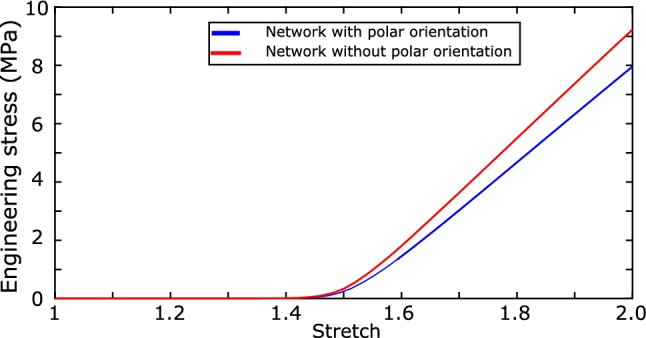
Comparison of collagen network behavior with and without the inclusion of polar angle

To further elucidate the significance of out-of-plane orientation of collagen on the mechanical response, a sample simulation was conducted. We include a phantom distribution for the polar angle distribution, which has an overall mean in the axial–circumferential plane, the likes of which have been studied briefly in the literature (Holzapfel et al. 2015; Weisbecker et al. 2015; Gasser et al. 2012). From Fig. 17, it could be concluded that the exclusion of polar angle has a noticeable effect. The stretch at which collagen was recruited was observed to be very slightly higher in the case of a 3-D oriented network, while the overall stiffness of the network in the loading direction slightly reduced. In short, as expected, the 3-D nature of orientation distributions, compared to 2-D, is the cause of a slightly higher magnitude of reorientation effects in uniaxial tension. Future studies, in particular experimental one involving the network behavior of collagen, should incorporate the measurement of out-of-plane orientation distribution if possible.

## Conclusions

The mechanical behavior of collagen microstructure under uniaxial tensile loading was studied with a discrete random line network model. The microstructural model incorporated relevant stochastic information of collagen fibers extracted from multi-photon microscopy image stacks. The underlying network kinematics and the global mechanical response were accurately predicted by the model. Reliable constitutive parameters corresponding to collagen and apparent transverse stiffness of the network were estimated by the model. From this investigation, the model was used to conclude important structure–function relationships that control the mechanical response. The present model, which corresponded to healthy aortas, can be extended to aneurysmal aortas in the future in light of improving our understanding of initiation and propagation of rupture. This model could be ultimately used to develop better safety measures in assessing the risk related to aortic rupture.
